# Pertussis

**Published:** 1863-02

**Authors:** 


*Pertussis.*—Dr. J. B. LUDEN (*Penn. Transac.*) recommends in hooping-cough the following mixture, which has proved to him the means both of alleviating, and cutting much shorter the whole disease: ℞—Ext. Belladonnæ, gr. iij; Infus. Ipecac ℥ iiiss; Sacch. Alb. ℥ iij; Aq. Amygdal ʒ ij—℥ ss. F. M. From half to one teaspoonful three or four times a day for children.

---

*Biennial Report of the Board of Trustees of the Michigan Asylum for the Insane*, 1862.—This Asylum is located at Kalamazoo, Mich., and is under the Superintendency of Dr. E. H. Van Deusen, formerly of the Utica (N. Y.,) Asylum. The report shows 155 patients under care Dec. 1st, 1862. This is the foundation of a model insane asylum, and under the wise and sagacious charge of Dr. Van Deusen, who is a model Superintendent, it is achieving a high reputation everywhere. We are exceedingly gratified to hear that the State Legislature has, the present session, appropriated $60,000 to enlarge the capacity of the asylum, and thus promote its further usefulness. The Peninsula State is to be congratulated.

